# Milk Metabolomics Reveals Potential Biomarkers for Early Prediction of Pregnancy in Buffaloes Having Undergone Artificial Insemination

**DOI:** 10.3390/ani10050758

**Published:** 2020-04-27

**Authors:** Donato de Nicola, Francesco Vinale, Angela Salzano, Giada d’Errico, Anastasia Vassetti, Nunzia D’Onofrio, Maria Luisa Balestrieri, Gianluca Neglia

**Affiliations:** 1 Department of Veterinary Medicine and Animal Production, University of Naples “Federico II”, 80137 Naples, Italy; denicolavet@gmail.com (D.d.N.); frvinale@unina.it (F.V.); neglia@unina.it (G.N.); 2 Institute for Sustainable Plant Protection, National Research Council, 80055 Portici (NA), Italy; 3 Department of Agricultural Sciences, University of Naples “Federico II”, 80055 Portici (NA), Italy; giada.derrico@unina.it (G.d.); an.vassetti@libero.it (A.V.); 4 Department of Precision Medicine, University of Campania Luigi Vanvitelli, 80138 Naples, Italy; nunzia.donofrio@unicampania.it (N.D.); MariaLuisa.BALESTRIERI@unicampania.it (M.L.B.)

**Keywords:** metabolome, milk, buffalo, artificial insemination, pregnancy, LC–MS, N-acetyl carnitine

## Abstract

**Simple Summary:**

Today, the ability to determine the pregnancy status of cows as soon as possible after artificial insemination (AI) has become the most important thing to obtain in an ideal farm. Several efforts have been made to discover biomarkers of early pregnancy but, as of today, without any particular result. Most of the studies were carried out on non-invasive and cheap biological fluids, such as milk. Therefore, in order to identify potential biomarkers of early pregnancy a metabolomic approach on milk of 10 pregnant and 10 non-pregnant buffaloes was used. Milk was recovered in different days before and after the AI, and the data were analyzed retrospectively. The results revealed significant differences between pregnant and non-pregnant buffaloes, as well as in the expression of five metabolites. These data suggest the effectiveness of the metabolomic analysis for the identification of novel potential biomarkers in early prediction of pregnancy in buffaloes after AI, and these findings would give breeders the opportunity to rebreed animals at the next estrus event, saving most of the days as open.

**Abstract:**

This study aimed to identify potential biomarkers for early pregnancy diagnosis in buffaloes subjected to artificial insemination (AI). The study was carried out on 10 pregnant and 10 non-pregnant buffaloes that were synchronized by Ovsynch-Timed Artificial Insemination Program and have undergone the first AI. Furthermore, milk samples were individually collected ten days before AI (the start of the synchronization treatment), on the day of AI, day 7 and 18 after AI, and were analyzed by LC–MS. Statistical analysis was carried out by using Mass Profile Professional (Agilent Technologies, Santa Clara, CA, USA). Metabolomic analysis revealed the presence of several metabolites differentially expressed between pregnant and non-pregnant buffaloes. Among these, a total of five metabolites were identified by comparison with an online database and a standard compound as acetylcarnitine (3-Acetoxy-4-(trimethylammonio)butanoate), arginine-succinic acid hydrate, 5′-O-{[3-({4-[(3aminopropyl)amino]butyl}amino)propyl]carbamoyl}-2′-deoxyadenosine, N-(1-Hydroxy-2-hexadecanyl)pentadecanamide, and N-[2,3-Bis(dodecyloxy)propyl]-L-lysinamide). Interestingly, acetylcarnitine was dominant in milk samples collected from non-pregnant buffaloes. The results obtained from milk metabolic profile and hierarchical clustering analysis revealed significant differences between pregnant and non-pregnant buffaloes, as well as in the metabolite expression. Overall, the findings indicate the potential of milk metabolomics as a powerful tool to identify biomarkers of early pregnancy in buffalo undergoing AI.

## 1. Introduction

Buffalo (*Bubalus bubalis*) breeding has achieved remarkable production standards in the last twenty years [1]. The competitiveness of breeding is further implemented by the use of reproductive biotechnologies, enabling selective objectives to be reached more quickly [2]. Among these, artificial insemination (AI) has been largely applied [3,4,5], reaching considerable results, comparable or higher than those obtained for cattle [6]. In fact, recently, the difficulties that lead to low fertility in buffaloes after AI have been largely overcome [6] through a deeper understanding of the physiological mechanisms that regulate the ovarian cycle [7], the factors that influence embryonic mortality [8,9], and the development of new protocols for the synchronization of estrus [6] and ovulation [10]. The latter two aspects are fundamental in buffaloes, since, in this specie, the estrus behavior is less evident and difficult to detect naturally, compared to cattle [11]. All these features allowed to gain a pregnancy rate higher than 50%.

The combined effect of consecutive AI treatments and the utilization of ultrasound is a reliable tool for reducing the days open, and consequently the intercalving period, increasing the reproductive efficiency of the herd in both pluriparous buffaloes [12] and heifers [13]. In fact, the possibility of utilizing some resynchronization protocols in non-pregnant buffaloes allows to reduce the interval between an unsuccessful AI and the subsequent insemination on the same animal [14]. However, one of the main limitations of these schedules is the time required for pregnancy detection, that can be hardly performed before 25 days post-mating [15]. For this reason, several attempts have been carried out to identify biomarkers of early pregnancy in cattle [16,17,18] but without satisfactory results. One of the first attempts that was developed to achieve an early pregnancy diagnosis was the detection of blood progesterone [19]. The latter is an indirect method for pregnancy and particularly non-pregnancy diagnosis in many livestock species including cattle, buffaloes, sheep, and goats [19,20,21]. In buffalo, the detection of plasma progesterone allows the diagnosis of non-pregnant animals after 21 days post AI [20], but it is reliable only if AI or breeding dates are known/recorded and has low accuracy due to the variable length of the estrus cycle. Some studies were carried out on pregnancy-associated glycoproteins (PAG), a large family of proteins that are produced by the placenta of ruminants [22,23]. Although the diagnosis through PAG assay is particularly reliable, it cannot be performed before 28 days of pregnancy [24]. In cattle, the potential of miRNA biomarkers for early pregnancy detection serving as a ‘biomarker signature’ in milk has been recently analyzed but without success for a diagnostic use [25].

The characterization of the metabolome can represent a new promising approach for identifying pregnant subjects. Metabolomics is a field of “omics” sciences very useful for the characterization of large numbers of small molecules within a biological sample using high-throughput methodologies [26,27]. Several techniques, such as liquid chromatography tandem-mass spectrometry (LC–MS/MS), nuclear magnetic resonance (NMR) spectroscopy and gas chromatography-mass spectrometry (GC-MS), have been developed and used for metabolite detection purposes [28,29,30]. The levels of these metabolites reflect the metabolic and health status of the subjects and may be used for discriminating among different phenotypes [31]. Evidence indicate that the presence or absence of specific combinations of milk metabolites is strongly correlated with its properties and the physiological status of animals [32]. Furthermore, some studies performed over the last 30 years demonstrated the fundamental importance of either metabolic hormones (such as growth hormone, insulin-like growth factor 1, insulin, leptin, and thyroxine) and metabolic factors (glucose, fatty acids) in the reproductive field [33].

Therefore, the aim of this study was to investigate the milk metabolomic profile for the identification of novel potential biomarkers to be used in early prediction of pregnancy in buffaloes that have undergone AI.

## 2. Materials and Methods

### 2.1. Farm and Animals

The trial was carried out on 20 animals (51.85 ± 0.72 days in milk and 2.95 ± 0.20 parity), selected in a large group of 31 buffaloes (125.90 ± 15.54 days in milk) during the transition period from decreasing to increasing day light length. The animals were bred in a commercial farm located in the South of Italy between 39.0° N and 41.5° N. Lactating buffaloes were maintained in open yards in 12 m^2^/head and 80 cm manger. Animals were fed a total mixed ration consisting of maize silage, oat hay, corn meal and soybean meal and characterized by 0.91 Milk Forage Units (MFU), 15% crude protein on dry matter, and 20% starch, with a forage concentrate ratio of 60:40. Buffaloes were milked twice daily, and milk yield was recorded in each milking by the software in the milking machine (Afifarm, TDM, Nutriservice, Brescia, Italy). The reproductive management of the herd was carried out by using a re-synch protocol, as described in another study [10]. At the beginning of the study, all buffaloes underwent clinical examination 12 days apart by clinical examination and ultrasound monitoring to evaluate the presence of a corpus luteum (CL) in at least one examination. Only buffaloes with a functional CL assessed by eco-color Doppler technique [34] and without any gross abnormality of the genital tract were included in the study.

### 2.2. Synchronization Treatment and AI

The buffaloes underwent synchronization of ovulation and timed artificial insemination by the Ovsynch-TAI protocol [5]. Briefly, it consists in a Gonadotropin-releasing hormone (GnRH) analogous administration on Day 0 (buserelin acetate, 12 mg; Receptal, Intervet, Milan, Italy), a prostaglandin F2α on Day 7 (Prostaglandin F_2α_ (PGF_2α_) analogue, luprostiol, 15 mg; Prosolvin, Intervet, Milan, Italy), and a further GnRH agonist administration on Day 9. Timed AI was performed on Day 10, at 60 and 16 h from the PGF_2α_ and the last GnRH, respectively. Because of the low intensity of estrus behavior in buffaloes, animals that showed a tonic uterus and a follicle higher than 1 cm, with or without mucus vaginal discharge, were considered to be in estrus and inseminated. Artificial inseminations were performed by the same technician using frozen/thawed semen of one bull of proven fertility.

Furthermore, on the day of AI each buffalo underwent an ultrasound examination with a portable machine (MyLab 30Gold, Esaote, Italy) equipped with a trans-rectal 7.5 MHz linear probe: preovulatory follicle (FL) dimensions were measured, and FL area was calculated according to the following equation:

(1) $$\mathrm{FL}\;\mathrm{area}=\left(\frac{a}{2}\right)\times \left(\frac{b}{2}\right)\times \pi ,$$

where: FL = Follicle; a = major axis of the follicle; b = minor axis of the follicle; π = 3.14.

### 2.3. Milk Sampling and Ultrasound Examination

Milk was sampled at the start of the synchronization treatment (Day −10; D −10), on the day of AI (Day 0; D0), 7 days after AI (+7; D7) and 18 days after AI (+18; D18). After collection milk was stored at −80 °C until analyses, that were performed after pregnancy assessment. Pregnancy diagnosis was performed on day 27 post-TAI using the same ultrasound machine described above and was confirmed on day 45 and 70 post-TAI. Animals that were pregnant on day 27 but not pregnant on day 45 were considered to have undergone late embryonic mortality (LEM), whereas those pregnant on day 45 but not on day 70 were considered to have experienced fetal mortality (FM). As described above, after pregnancy diagnosis assessment, 20 pluriparous buffaloes (between second and third parity) that have undergone the first AI (10 pregnant and 10 non-pregnant) were selected. The differences between pregnant and non-pregnant subjects were retrospectively analyzed by a metabolomic approach.

### 2.4. Metabolomic Analysis

The milk metabolites extraction was carried out mixing 100 μL from each sample with 300 µL of methanol (Sigma-Aldrich, Milan, Italy) and vortexing for 30 s at room temperature. Mixtures were centrifuged at 12,000× *g* for 15 min at 4 °C. Then, extracted supernatants were used. LC–MS analyses for detection of milk metabolites and for acetyl carnitine (3-Acetoxy-4-(trimethylammonio)butanoate) identification and quantification were done using an HPLC 1260 Infinity Series (Agilent Technologies, Santa Clara, CA, USA) coupled to a Q-TOF mass spectrometermodel G6540B (Agilent Technologies) with a Dual Electrospray Ionization (ESI) source and equipped with a Diode Array Detector (DAD) system (Agilent Technologies). Ascentis^®^ Express C18 column (2.7 μm, 50 mm × 3.0 mm i.d., Supelco^©^, Bellefonte, PA, USA) was used for separations. Flow-rate was set at 0.500 mL/min. The elution was done at constant temperature of 40 °C, using a linear gradient composed by A: 0.1% (*v/v*) formic acid (FA) in H_2_O and B: 0.1% (*v/v*) FA in acetonitrile (ACN). The gradient was as follows: starting condition 5% B, ramping to 95% B in 12 min, lowering to 5% B in 1 min and equilibration at 5% B for 5 min. UV spectra were collected by DAD every 0.4 s from 190 to 750 nm with a resolution of 2 nm. Targeted MS and targeted tandem-mass spectrometry (MS/MS) parameters were set with Agilent MassHunter Data Acquisition Software, rev. B.05.01. The instrument operated in positive mode, as [M + H] + ions; MS spectra were recorded in centroid mode, with an m/z 50–1700 mass range and with a speed of 3.3 spectra/s. Capillary voltage was set at 2000 V, fragmentor at 180 V, cone 1 (skimmer 1) at 45 V, Oct RFV at 750 V. Drying gas flow was set at 11L/min at a temperature of 350 °C, and the nebulizer was set at 45 psig. The injected sample volume was 5 μL.

In order to perform real-time lock mass correction, an Isocratic pump (1260 Infinity Series, Agilent Technologies) was used to infuse a standard solution consisting of two reference mass compounds: purine (C_5_H_4_N_4_, m/z 121.050873, 10 μmol/L) and hexakis (1H,1H,3H-tetrafluoropentoxy)-phosphazene (C_18_H_18_O_6_N_3_P_3_F_24_, m/z 922.009798, 2 μmol/L). Flow rate was set at 0.06 mL/min, while the detection window and the minimum height were set at 1000 ppm and 10,000 counts, respectively, for reference mass correction.

### 2.5. Metabolite Identification and Quantification

Raw data were evaluated using Mass Hunter Qualitative Analysis Software, rev B.06.00 (Agilent Technologies, Santa Clara, CA, USA, while compound identification was carried out using a freely available electronic database Milk Composition Database (MCDB) and an in-house database. N-acetyl carnitine was quantified by comparison with a standard compound purchased from Sigma-Aldrich.

### 2.6. Statistical Analyses

Data on milk yield and follicle area between pregnant and non-pregnant buffaloes were analyzed by ANOVA. Statistical analysis of metabolomic data was carried out by using Mass Profile Professional, version 13.1.1 (Agilent Technologies). Specifically, a one-way analysis of variance, one-way ANOVA (*p*-value < 0.05), was performed and results obtained were subjected to principal components analysis (PCA) and hierarchical clustering.

## 3. Results

### 3.1. Reproductive Activity

Total pregnancy rate detected 27 days after AI was 54.8% (17/31) and declined to 45.2% (14/31) on day 45, which means an incidence of late embryonic mortality (LEM) of 17.6% (3/17). No fetal mortality was recorded during this investigation. Further data are reported only for 20 selected buffaloes. No differences were found on milk yield between pregnant and non-pregnant buffaloes (Table 1).

The size of the preovulatory follicle recorded on the day of Ovsynch-TAI protocol (TAI) was significantly (*p* < 0.05) lower in pregnant buffaloes than non-pregnant counterparts (1.21 ± 0.1 vs. 1.40 ± 0.1 cm, respectively).

### 3.2. Milk Metabolic Profile

**Figure 1:**
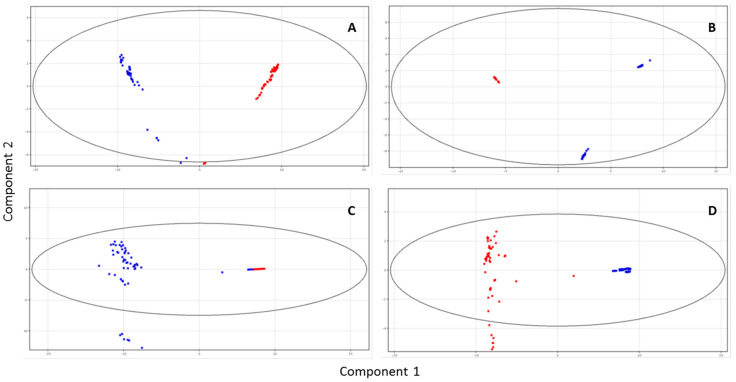
Principal components analysis (PCA) scores plots of the LC–MS data acquired for the four milk samplings from pregnant (in red) and non-pregnant (in blue) buffaloes. (**A**) (first sampling): PC1 occupies 69.37% and PC2 7.42% of total variance; (**B**) (second sampling): PC1 72.26% and PC2 27.23%; (**C**) (third sampling): PC1 61.45% and PC2 7.04%; and (**D**) (fourth sampling): PC1 79.85% and PC2 2.83%.

The milk metabolic profile differed between pregnant buffaloes and those non-pregnant. Distinct separation between these two groups was evident in principal components (PC1–PC2) of the variance in the LC–MS dataset. More in detail, PC1–PC2 values were accounting 76.79–99.49% and 68.49–82.68%, respectively, for each sample (Figure 1A–D).

**Figure 2:**
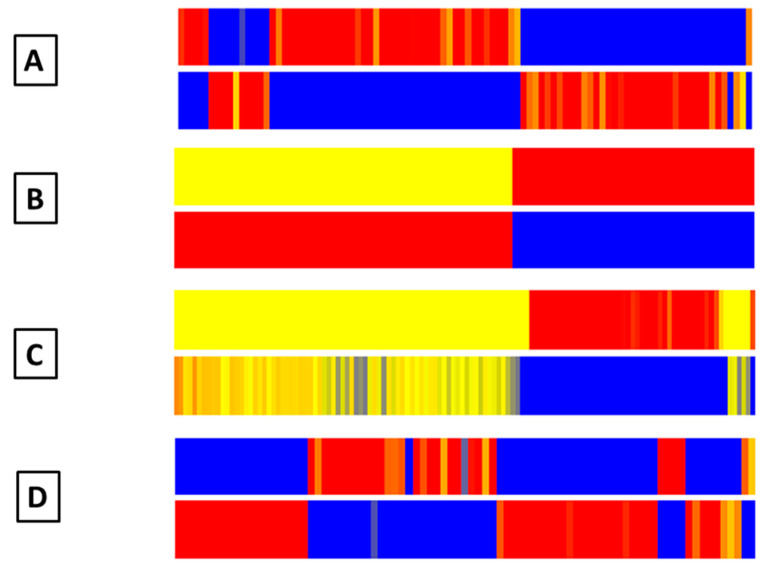
Hierarchical clustering of 80 ((**A**) first sampling), 67 ((**B**) second sampling), 103 ((**C**) third sampling), and 81 ((**D**) fourth sampling) differentially expressed compounds in pregnant (P) and non-pregnant (NP) buffaloes. The differently expressed compounds are reported in Tables S1–S4.

Moreover, hierarchical clustering analysis also showed a clear separation of pregnant and non-pregnant buffaloes. Based on the PCA loadings, a list of metabolites (Tables S1–S4, reported in supplementary materials) whose changes in milk led to the clustering is presented in Figure 2A–D). Clustering for the two groups of buffaloes highlighted the total variances within the PCA data. Differences between pregnant and non-pregnant buffaloes were clearly associated with differences in chemical composition (Figure 2).

Metabolomic analysis data revealed the presence of several metabolites differentially expressed (80, 67, 103, and 81 at D −10, D0, D7, and D18, respectively; Tables S1–S4) in the milk of pregnant and non-pregnant buffaloes. Among these, five metabolites were identified by comparison with an online database and a standard compound. The identified compounds were arginine-succinic acid hydrate, 5′-O-{[3-({4-[(3Aminopropyl)amino]butyl}amino)propyl]carbamoyl}-2′-deoxyadenosine, N-(1-Hydroxy-2-hexadecanyl) pentadecanamide, N-[2,3-Bis(dodecyloxy)propyl]-L-lysinamide), and acetyl carnitine (Table 2).

This metabolomic profile was dominated by the presence of acetyl carnitine. Targeted analysis of N-acetyl carnitine revealed its occurrence in milk samples from non-pregnant buffaloes starting from D −10 up to D18, with a consistent decrease over the sampling time (Table 3). On the contrary, in milk from pregnant buffaloes, acetyl carnitine was observed only in the last sampling time (D18) (Table 3).

## 4. Discussion

The improvement of reproductive efficiency is a key point to reduce the intercalving period and optimize the economic sustainability of the buffalo farm. This is particularly important in Italy, in order to guarantee milk availability during the period of greatest market demand for mozzarella cheese production [35]. The application of re-synchronization protocols, together with eco Color-Doppler technique, allowed to gain an intercalving period of about 400 days [12]. However, a reliable pregnancy diagnosis can be carried out at least 25 days post-AI by ultrasound, limiting the efficiency of the technique and increasing the number of days open. This can be achieved by both identifying real potential biomarkers of early pregnancy, such as those compounds that differ between pregnant and non-pregnant animals on D7 or D18 and recording different molecules before insemination. In fact, on D7 and D18 a pregnancy is effectively ongoing, whereas differences in milk metabolites on D −10 and D0 may account for the incapacity of conception, i.e., for the development of the preovulatory follicle: therefore, they can be defined as biomarkers of potential conception.

The pregnancy rate recorded in this trial is comparable to that obtained in other studies, as well as the incidence of LEM [12,36]. The latter is considered one of the main causes of fertility loss, in particular in some periods of the year [8,9]. Both the seasonality of the species and the market requirements of milk in Italy, oblige to mate the animals out of the breeding season, increasing the incidence of LEM till 40% in some farms [37]. On the contrary, an incidence of LEM higher than 10% is rarely recorded during the breeding season [38,39].

According to our results, the diameter of the preovulatory follicle may play a key role in the improvement of AI efficiency. In fact, a significantly smaller diameter has been recorded in pregnant buffaloes compared to non-pregnant counterparts. Several studies carried out in cattle demonstrated the deleterious effects of a prolonged follicle dominance on oocyte competence and embryo development [40]. It is likely that, particularly in animals synchronized by the Ovsynch-TAI Program, the failure of ovulation following the first GnRH administration may be responsible for a persistent dominant follicle [41]. This interesting aspect demonstrated in cattle [42] may be confirmed by a recent trial carried out on buffalo species [5], in which the follicular response to the first GnRH of an Ovsynch-TAI program on pregnancy outcome was evaluated. In this study buffaloes that ovulated after the administration of the first GnRH showed smaller area of the ovulatory follicle compared to those that did not ovulate. Interestingly, these animals had also a significantly greater ovulation rate to the second GnRH [5]. Further studies are needed to evaluate this aspect in buffalo.

The main aim of this study was to identify potential biomarkers for early pregnancy diagnosis in buffalo through a metabolomic approach. Over the last years, this technique was tested in several fields of veterinary science, using different biological fluids [43,44,45]. In dairy animals the milk represents an ideal substrate, as the procedure for collection is easy, non-invasive and without any stress for the animals. Blood metabolites are concentrated in the milk by filtration, thus reflecting the metabolic status of the subjects. To our knowledge this is the first study that utilizes LC–MS technique for the identification of novel biomarker of early pregnancy in buffalo milk.

Several pathways involved in the milk biosynthesis were different between pregnant and non-pregnant buffaloes in this study. Milk metabolite profiling has been found to be successfully related to the onset of early pregnancy after AI treatment.

Among the compounds differently expressed, only acetyl carnitine was quantified. Interestingly, this compound was present in milk samples from non-pregnant buffaloes collected in each sampling time, while only on D18 was detected in pregnant animals. Likely, the source of acetyl carnitine in the milk is relocated or lost in favor of other tissues.

Metabolic analysis in early pregnancy is crucial for the identification of molecular pathways useful for the definition of new treatment strategies Among metabolites in milk from pregnant buffaloes, a positive modulation was observed for N-(1-Hydroxy-2-hexadecanyl) pentadecanamide (D10, D0), arginine- succinic acid hydrate (D10, D0), and acetyl carnitine (D18). Instead, unchanged or negatively modulated metabolites of nutritional value were 5′-O-{[3-({4-[(3 Aminopropyl)amino]butyl}amino)propyl]carbamoyl}-2′-deoxyadenosine, and N-[2,3-Bis(dodecyloxy)propyl]-L-lysinamide.

N-(1-Hydroxy-2-hexadecanyl) pentadecanamide is a derivative of pentadecanoic acid, a fatty acid with exogenous origin (primarily ruminant), which constitutes 1.05% of milk fat and 0.43% of ruminant meat fat. It belongs to the odd chain saturated fatty acids (OCS-FAs) family and is produced in relatively high levels by rumen microbial fermentation and microbial de-novo lipogenesis, which then transfers into the host animal [46]. Moreover, the OCS-FAs produced by the animal rumen are then utilized by the mammary gland for the production of milk fat [47]. Increased consumption of dairy products has been associated with an increase of OCS-FAs plasma levels. Indeed, pentadecanoic acid has been gaining interest within the scientific community as it has been found to be important as low-cost internal standards in quantitative analysis, a blood biomarker for milk fat intake, and biomarker for coronary heart disease [48].

Acetylcarnitine from food source or as a supplement is known for its antioxidant activity, with particular regard to the neuroprotective action against central and peripheral nervous system injury [49,50,51].

Arginine- succinic acid hydrate is an arginine derivative. L-Arginine, is known to be particularly abundant in certain foods, such as meats and nuts, is the substrate for the enzyme nitric oxide synthase (NOS), involved in the production of nitric oxide (NO) [52,53]. Recently, arginine-rich foods were shown to be inversely associated with endothelial dysfunction in hypercholesterolemia patients. In particular, evidence show that long-term L-arginine intake increases insulin sensitivity, improves glycemic indices, and reduces cardiovascular complications [54,55,56].

5′-O-{[3-({4-[(3Aminopropyl)amino]butyl}amino)propyl]carbamoyl}-2′-deoxyadenosine is an adenosine derivative. Adenosine, an essential metabolite distributed in several mammalian tissues [57,58], acts as a ubiquitous endogenous cell signaling and modulator agent since it directly affects a variety of synaptic processes and signaling pathways, thus playing an important role in the regulation of several neurotransmitters in the central nervous system [59]. Moreover, adenosine produced in hypoxic, ischemic, or inflamed environments reduces tissue injury and promotes repair [60]. Due to its extremely short half-life in human blood, prolonged release systems to improve its efficacy or food source capable of sustaining its intake are of great interest [61,62,63,64].

Finally, our metabolomic analysis showed unchanged levels of *N*-[2,3-Bis(dodecyloxy)propyl]-l-lysinamide from D −10 up to D7. This metabolite is a derivative of lysine, an essential amino acid commonly assumed as dietary supplement, especially in countries where insufficient L-lysine is ingested from food. Indeed, supplemental L-lysine benefits include increase in the intestinal calcium absorption in osteoporosis, anxiety reduction, and muscle recovery in exercise or sarcopenia support [65,66,67].

## 5. Conclusions

On the whole, findings of this study reveal a peculiar metabolic profile of milk produced by pregnant and non-pregnant buffaloes with significant differences in their content. Beside the chemical and nutritional characterization of milk, these data suggest the effectiveness of the metabolomic analysis for the identification of novel potential biomarkers in early prediction of pregnancy in buffaloes after AI.

## Figures and Tables

**Table 1:** Average of milk yield (kg) in successfully pregnant (P) and non-pregnant (NP) buffaloes on Day 70 post artificial insemination (AI), measured at the beginning of the synchronization treatment (Day −10), at the artificial insemination (Day 0), and later at 7 (+7) and 18 days (+18).

Groups	Time (Days)			
	−10	0	+7	+18
P	8.78 ± 0.9	8.15 ± 0.9	9.02 ± 1.0	8.25 ± 0.8
NP	7.48 ± 0.8	6.88 ± 0.9	7.43 ± 0.8	7.49 ± 0.8

Data are expressed as means ± standard error.

**Table 2:** Secondary metabolites identified in the milk samples. Identifications were confirmed by comparing results with known compounds present in a freely available electronic database Milk Composition Database (MCDB), an in-house database/standards and selecting matching, with a score ≥95%.

Metabolites	Sampling Times			
	D −10	D0	D7	D18
Acetyl carnitine (3-Acetoxy-4-(trimethylammonio)butanoate)	↓	↓	↓	↑
Arginine-succinic acid hydrate	↑	↑	↔	↔
5′-O-{[3-({4-[(3 Aminopropyl)amino]butyl}amino)propyl]carbamoyl}-2′-deoxyadenosine	↔	↓	↓	↔
N-(1-Hydroxy-2-hexadecanyl) pentadecanamide	↑	↑	↔	↔
N-[2,3-Bis(dodecyloxy)propyl]-L-lysinamide	↔	↔	↔	↓

↑ Increased production of the metabolite in pregnant vs. non-pregnant. ↓ Decreased production of the metabolite in pregnant vs. non-pregnant. ↔ Unchanged production of the metabolite in pregnant vs. non-pregnant.

**Table 3:** N-acetyl carnitine (g/mL) quantification recorded in successfully pregnant (P) and non-pregnant (NP) buffaloes on Day 70 post AI, measured at the beginning of the synchronization treatment (Day −10), at the artificial insemination (Day 0), and later at 7 (+7) and 18 days (+18).

Group	N-acetyl Carnitine (g/mL)			
	Sampling Times			
	D −10	D0	D7	D18
P	N.D.	N.D.	N.D.	0.3 ± 0.5
NP	2.1 ± 1.1	3.3 ± 3.2	0.8 ± 0.5	0.2 ± 0.4

N.D. = Not detectable. Data are expressed as means ± ES.

## Supplementary Materials

The following are available online at https://www.mdpi.com/2076-2615/10/5/758/s1, Table S1. Secondary metabolites differentially expressed in pregnant and non-pregnant buffaloes at D −10; Table S2. Secondary metabolites differentially expressed in pregnant and non-pregnant buffaloes at D0; Table S3. Secondary metabolites differentially expressed in pregnant and non-pregnant buffaloes at D7; Table S4. Secondary metabolites differentially expressed in pregnant and non-pregnant buffaloes at D18.
