# Supplementary material for: Milk Metabolomics Reveals Potential Biomarkers for Early Prediction of Pregnancy in Buffaloes Having Undergone Artificial Insemination
Source: Animals (Basel). 2020 Apr 27;10(5):758. doi: 10.3390/ani10050758 (PMC7277816; doi:10.3390/ani10050758)
Supplement: Supplementary file 1 [file animals-10-00758-s001.pdf]

animals

MDPI

Article

Milk Metabolomics Reveals Potential Biomarkers for Early Prediction of Pregnancy in Buffaloes Having Undergone Artificial Insemination

Donato de Nicola [1], Francesco Vinale [1,2], Angela Salzano [1,*], Giada d'Errico [3], Anastasia Vassetti [3], Nunzia D'Onofrio [4], Maria Luisa Balestrieri [4] and Gianluca Neglia [1]

1 Department of Veterinary Medicine and Animal Production, University of Naples "Federico II", 80137 Naples, Italy; denicolavet@gmail.com (D.D.N.); frvinale@unina.it (F.V.); neglia@unina.it (G.N.)
2 Institute for Sustainable Plant Protection, National Research Council, 80055 Portici (NA), Italy
3 Department of Agricultural Sciences, University of Naples "Federico II", 80055 Portici (NA), Italy; giada.derrico@unina.it (G.D.); an.vassetti@libero.it (A.V.)
4 Department of Precision Medicine, University of Campania Luigi Vanvitelli, 80138 Naples, Italy; nunzia.donofrio@unicampania.it (N.D.O.); MariaLuisa.BALESTRIERI@unicampania.it (M.L.B.)
* Correspondence: angela.salzano@unina.it; Tel.: +39 0812536215

SUPPLEMENTARY MATERIALS

Table 1. Secondary metabolites differentially expressed in pregnant and non-pregnant buffaloes at D10 (Figure 2 A).

Table 2. Secondary metabolites differentially expressed in pregnant and non-pregnant buffaloes at D0 (Figure 2B).

Table 3. Secondary metabolites differentially expressed in pregnant and non-pregnant buffaloes at D7 (Figure 2C).

Table 4. Secondary metabolites differentially expressed in pregnant and non-pregnant buffaloes at D18 (Figure 2D).

Table 1. Secondary metabolites differentially expressed in pregnant and non-pregnant buffaloes at D10 (Figure 2A).

Compound	Mass
Acetyl carnitine (3-Acetoxy-4-(trimethylammonio)butanoate)	203.1147
$C_{8} H_{21} N_{5} O$	203.1738
$C_{17} H_{37} N O_{2}$	287.2821
$C_{14} H_{16} N6 O_{2}$	300.1325
$C_{17} H_{26} O_{5}$	310.1766
$C_{19} H_{34} O_{3}$	310.2505
$C_{15} H_{24} N_{7} O_{3}$	350.1935
$C_{12} H_{22} O_{11}$	364.0980
$C_{14} H_{28} N_{4} O_{5} S$	364.1771
Arginine - succinic acid hydrate	364.1805
$C_{13} H_{12} N_{6} O_{8}$	380.0715
$C_{11} H_{22} N_{7} O_{8}$	380.1534
$C_{15} H_{16} N_{3} O_{7} S$	382.0708
$C_{12} H_{28} Cl_{2} N_{8} O_{2}$	386.1709
$C_{19} H_{38} N_{4} S_{2}$	386.2539
$C_{16} H_{40} N_{5} O_{6}$	398.2991
$C_{19} H_{38} N_{6} O_{3}$	398.2992
$C_{19} H_{40} N_{9} O_{3}$	442.3250
$C_{21} H_{42} N_{6} O_{4}$	442.3253

$C_{24}H_{52}N_5O_2$	442.4121
$C_{22}H_{44}N_6O_4$	456.3406
$C_{20}H_{42}N_9O_3$	456.3410
$C_{23}H_{52}N_8O$	456.4295
$C_{25}H_{41}N_3O_6$	479.2992
$C_{18}H_{47}N_{12}O_3$	479.3898
$C_{21}H_{51}N_7O_5$	481.3958
$C_{24}H_{49}N_8O_2$	481.3959
N-(1-Hydroxy-2-hexadecanyl) pentadecanamide	481.4858
$C_{20}H_{48}N_5O_8$	486.3515
$C_{26}H_{58}N_6S$	486.4432
$C_{26}H_{45}N_3O_6$	495.3304
$C_{22}H_{49}N_{13}$	495.4231
$C_{29}H_{63}N_6O$	511.5001
$C_{26}H_{45}N_6O_5$	521.3458
$C_{30}H_{57}N_4O_3$	521.4408
$C_{22}H_{49}N_{14}O$	525.4215
$C_{26}H_{53}N_8O_3$	525.4215
$C_{23}H_{55}N_7O_6$	525.4220
$C_{33}H_{69}N_2S$	525.5197
$C_{22}H_{52}N_5O_9$	530.3771
$C_{27}H_{60}N_7O_3$	530.4744
$C_{31}H_{67}N_6O$	539.5337
$C_{34}H_{71}N_2S$	539.5339
$C_{31}H_{67}N_6O_2$	555.5318
$C_{17}H_{37}N_7O_{14}$	563.2401
$C_{23}H_{47}N_8O_8$	563.3520
$C_{24}H_{46}N_3O_{12}$	568.3074
$C_{24}H_{53}N_{14}O_2$	569.4473
$C_{28}H_{57}N_8O_4$	569.4474
$C_{32}H_{69}$ N6 O_2	569.5490
$C_{24}H_{56}N_5O_{10}$	574.4028
$C_{24}H_{46}N_{16}O$	574.4029
$C_{33}H_{71}N_6O_2$	583.5639
$C_{35}H_{73}N_3O_3$	583.5643
$C_{27}H_{50}N_4O_{10}$	590.3523
$C_{33}H_{71}N_6O_3$	599.5608
$C_{18}H_{39}N_8O_{15}$	607.2523
$C_{27}H_{53}N_5O_{10}$	607.3786
$C_{28}H_{59}N_{11}O_4$	613.4737
$C_{38}H_{77}O_5$	613.5773
$C_{39}H_{73}N_4O$	613.5773
$C_{25}H_{54}N_{12}O_6$	618.4295
$C_{20}H_{37}N_{13}O_{12}$	651.2650
$C_{29}H_{57}N_5O_{11}$	651.4045
$C_{28}H_{54}N_3O_{14}$	656.3609
$C_{40}H_{83}NO_3S$	657.6069
$C_{38}H_{81}N_4O_2S$	657.6074
$C_{30}H_{55}N_{12}O_7$	695.4308
$C_{39}H_{83}N_5O_5$	701.6356
$C_{22}H_{38}N_6O_{20}$	706.2141
$C_{25}H_{50}N_6O_{17}$	706.3231
*	223.6216
*	330.3534
*	495.5052
*	627.5956
*	643.5908
*	1990.5967

*	2235.7114
*	3980.1743
*	4481.3916

*compounds for which the Software Mass Profile Professional. version 13.1.1 (Agilent Technologies) did not generate the molecular formula.

Table 2. Secondary metabolites differentially expressed in pregnant and non-pregnant buffaloes at D0 (Figure 2B).

Compound	Mass
$C_6 H_9 N_3$	123.0782
Acetyl carnitine (3-Acetoxy-4-(trimethylammonio)butanoate)	203.1147
$C_{17} H_{32} N_3 O_2$	310.2493
$C_9 H_{20} N_2 O_{13}$	364.0957
$C_{10} H_{24} N_{10} O_3 S$	364.1758
Arginine-succinic acid hydrate	364.1805
$C_{17} H_{40} N_6 O_3$	376.3151
$C_{11} H_{10} N_9 O_7$	380.0693
$C_{13} H_{12} N_6 O_8$	380.0719
$C_{10} H_{26} N_3 O_{12}$	380.1504
$C_{20} H_{43} N_{11}$	437.3671
$C_{22} H_{50} N_8 O$	442.4115
$C_{20} H_{49} N_7 O_4$	451.3837
$C_{21} H_{45} N_{11} O$	467.3785
5'-O-{[3-({4-[(3 Aminopropyl)amino]butyl}amino)propyl]carbamoyl}-2'-deoxyadenosine	479.2967
$C_{20} H_{45} N_{14}$	481.3939
N-(1-Hydroxy-2-hexadecanyl)pentadecanamide	481.4858
$C_{21} H_{47} N_{14}$	495.4089
$C_{22} H_{49} N_{13}$	495.4216
$C_{23} H_{49} N_{11} O_2$	511.4033
$C_{19} H_{41} N_{11} O_6$	519.3266
$C_{24} H_{51} N_{11} O_2$	525.4192
$C_{36} H_{65} N_2$	525.5159
$C_{30} H_{64} N_3 O_2 S$	530.4711
$C_{23} H_{51} N_{14} O$	539.4344
$C_{34} H_{71} N_2 S$	539.5324
$C_{23} H_{51} N_{14} O_2$	555.4318
$C_{20} H_{39} N_{18} O_2$	563.3533
$C_{24} H_{53} N_{14} O_2$	569.4462
$C_{32} H_{69} N_6 O_2$	569.5454
$C_{29} H_{64} N_7 O_4$	574.5012
$C_{25} H_{55} N_{14} O_2$	583.4629
$C_{33} H_{71} N_6 O_2$	583.5625
$C_{35} H_{73} N_3 O_3$	583.5650
$C_{27} H_{57} N_{11} O_4$	599.4550
$C_{18} H_{39} N_8 O_{15}$	607.2532
$C_{23} H_{49} N_{11} O_8$	607.3793
$C_{28} H_{59} N_{11} O_4$	613.4725
$C_{38} H_{77} O_5$	613.5751
$C_{38} H_{79} N O_2 S$	613.5784
$C_{27} H_{59} N_{14} O_3$	627.4862
$C_{25} H_{57} N_{17} O_3$	643.4808
$C_{24} H_{47} N_{18} O_4$	651.3994
$C_{38} H_{81} N_4 O_2 S$	657.6049
$C_{41} H_{77} N_4 O_2$	657.6049
$C_{37} H_{79} N_5 O_4$	657.6083
$C_{27} H_{61} N_{17} O_3$	671.5122
$C_{29} H_{63} N_{14} O_5$	687.5060

$C_{26} H_{51} N_{18} O_5$	695.4307
$C_{29} H_{59} N_{21}$	701.5234
$C_{22} H_{38} N_6 O_{20}$	706.2102
$C_{30} H_{61} N_{21} O$	731.5362
$C_{29} H_{61} N_{24}$	745.5504
*	223.6216
*	330.3534
*	495.5026
*	495.5052
*	511.5008
*	555.5285
*	599.5585
*	627.5956
*	643.5908
*	662.4501
*	687.5083
*	695.4313
*	706.3176
*	1226.4552

*compounds for which the Software Mass Profile Professional. version 13.1.1 (Agilent Technologies) did not generate the molecular formula.

Table 3. Secondary metabolites differentially expressed in pregnant and non-pregnant buffaloes at D7 (Figure 2C).

Compound	Mass
$C_6 H_9 N_3$	123.0783
Acetyl carnitine (3-Acetoxy-4-(trimethylammonio)butanoate)	203.1147
$C_8 H_{21} N_5 O$	203.1727
$C_{13} H_{22} N_6 O_3$	310.1753
$C_9 H_{30} N_{10} O_2$	310.2489
$C_{19} H_{44} N_3 O$	330.3508
$C_{20} H_{41} N_4$	337.3304
$C_9 H_{20} N_2 O_{13}$	364.0954
$C_{16} H_{28} O_9$	364.1734
$C_{10} H_{24} N_{10} O_3 S$	364.1754
$C_{10} H_{14} N_5 O_{11}$	380.0683
$C_{10} H_{26} N_3 O_{12}$	380.1499
$C_{17} H_{45} N_{10} O_3$	437.3670
$C_{16} H_{42} N_8 O_6$	442.3218
$C_{15} H_{36} N_{15} O$	442.3228
$C_{22} H_{50} N_8 O$	442.4096
$C_{31} H_{54} O$	442.4104
$C_{18} H_{47} N_{10} O_3$	451.3824
$C_{16} H_{38} N_{15} O$	456.3382
$C_{23} H_{52} N_8 O$	456.4279
$C_{25} H_{54} N_5 O_2$	456.4280
$C_{20} H_{39} N_5 O_8$	477.2810
5'-O-{[3-({4-[(3 Aminopropyl)amino]butyl}amino)propyl]carbamoyl}-2'-deoxyadenosine	479.2967
$C_{18} H_{49} N_{13} S$	479.3872
$C_{17} H_{47} N_{13} O_3$	481.3935
$C_{19} H_{49} N_{10} O_4$	481.3935
$C_{29} H_{61} N_4 O$	481.4842
$C_{28} H_{61} N_6$	481.4844
$C_{17} H_{40} N_{15} O_2$	486.3482
$C_{29} H_{54} N_6$	486.4402
$C_{23} H_{54} N_{10} O$	486.4405
$C_{18} H_{49} N_{13} O_3$	495.4087

$C_{27} H_{55} N_6 S$	495.4202
$C_{29} H_{63} N_6$	495.5015
$C_{19} H_{48} N_8 O_7$	500.3634
$C_{17} H_{46} N_{11} O_6$	500.3636
$C_{18} H_{49} N_{13} O_4$	511.4030
$C_{29} H_{63} N_6 O$	511.4984
$C_{33} H_{55} N_5$	521.4381
$C_{20} H_{47} N_{17}$	525.4192
$C_{32} H_{67} N_3 O_2$	525.5143
$C_{36} H_{65} N_2$	525.5145
$C_{34} H_{69} O_3$	525.5147
$C_{17} H_{42} N_{18} O_2$	530.3744
$C_{30} H_{64} N_3 O_2 S$	530.4711
$C_{21} H_{49} N_{17}$	539.4334
$C_{35} H_{71} O_3$	539.5312
$C_{19} H_{50} N_{11} O_7$	544.3885
$C_{19} H_{47} N_{20}$	555.4282
$C_{38} H_{69} N O$	555.5342
$C_{20} H_{49} N_{20}$	569.4451
$C_{32} H_{69} N_6 O_2$	569.5444
$C_{34} H_{73} N_4 S$	569.5519
$C_{21} H_{51} N_{20}$	583.4593
$C_{35} H_{75} N_4 S$	583.5607
$C_{20} H_{48} N_{18} O_3$	588.4149
$C_{21} H_{51} N_{20} O$	599.4550
$C_{19} H_{25} N_{23} O_2$	607.2541
$C_{22} H_{53} N_{20} O$	613.4705
$C_{34} H_{73} N_6 O_3$	613.5733
$C_{35} H_{75} N_5 O_3$	613.5754
$C_{23} H_{55} N_{20} O$	627.4847
$C_{21} H_{53} N_{23} O$	643.4803
$C_{20} H_{53} N_{26}$	657.4966
$C_{40} H_{77} N_6 O$	657.6028
$C_{41} H_{77} N_4 O_2$	657.6042
$C_{21} H_{52} N_{21} O_4$	662.4512
$C_{28} H_{63} N_{17} O_4$	701.5216
$C_{26} H_{36} N_5 O_{18}$	706.2067
$C_{28} H_{38} N_2 O_{19}$	706.2090
$C_{21} H_{46} N_{12} O_{15}$	706.3182
$C_{42} H_{73} N_4 O7$	745.5475
*	495.5050
*	563.3520
*	574.5010
*	607.3780
*	364.0976
*	398.1677
*	398.2968
*	398.3796
*	398.3831
*	467.3766
*	467.4712
*	495.3272
*	495.5054
*	519.3253
*	530.4747
*	544.3891
*	583.5621
*	618.4254

*	618.5341
*	627.5951
*	643.5914
*	662.4501
*	687.5083
*	695.4313
*	706.3176
*	1226.4552
*	2235.7092
*	2236.2046
*	2236.5254
*	3980.1887
*	3980.7107

*compounds for which the Software Mass Profile Professional. version 13.1.1 (Agilent Technologies) did not generate the molecular formula.

Table 4. Secondary metabolites differentially expressed in pregnant and non-pregnant buffaloes at D18 (Figure 2D).

Compound	Mass
$C_6 H_9 N_3$	123.0791
Acetyl carnitine (3-Acetoxy-4-(trimethylammonio)butanoate)	203.1147
$C_{17} H_{37} N O_2$	287.2828
$C_{17} H_{26} O_5$	310.1774
$C_{17} H_{32} N_3 O_2$	310.2518
$C_{17} H_{36} N_3 O_3$	330.2766
$C_{22} H_{43} N O$	337.3339
$C_9 H_{20} N_2 O_{13}$	364.0982
$C_{14} H_{28} N_4 O_5 S$	364.1771
$C_{18} H_{10} N_8 O_2$	370.0932
$C_{19} H_{42} N_3 O_4$	376.3191
$C_{13} H_{24} N_4 O_9$	380.1535
$C_{18} H_{40} N_2 O_6$	380.2886
$C_{15} H_{38} Cl_2 N_7$	386.2559
$C_{18} H_{38} N_2 O_7$	394.2677
$C_{17} H_{44} N_7 O_3$	394.3516
$C_{21} H_{49} N_4 O_5$	437.372
$C_{20} H_{46} N_2 O_8$	442.3271
$C_{25} H_{54} N_5 O_2$	456.4307
$C_{18} H_{39} N_9 O_4 S$	477.2844
$C_{18} H_{41} N_9 O_4 S$	479.3005
$C_{25} H_{49} N_7 O_2$	479.3916
$C_{18} H_{49} N_{13} S$	479.3944
$C_{18} H_{43} N_9 O_4 S$	481.3161
$C_{23} H_{53} N_4 O_6$	481.3979
$C_{28} H_{61} N_6$	481.4879
$C_{16} H_{46} N_{12} O_3 S$	486.3516
$C_{21} H_{52} N_{13}$	486.4438
$C_{26} H_{58} N_6 S$	486.4444
$C_{14} H_{39} N_{16} O_4$	495.3321
$C_{24} H_{51} N_{10} O$	495.4251
$C_{29} H_{63} N_6 O$	511.5022
$C_{21} H_{53} N_{13} S$	519.4268
$C_{28} H_{47} N_3 O_6$	521.3475
$C_{30} H_{57} N_4 O_3$	521.4437
$C_{24} H_{51} N_{11} O_2$	525.4229
$C_{30} H_{65} N_6 O$	525.5183
$C_{31} H_{67} N_6 O$	539.5352

N-[2.3-Bis(dodecyloxy)propyl]-L-lysinamide	555.5336
$C_{18} H_{19} N_{14} O_8$	559.1506
$C_{17} H_{39} N_{10} O_9 S$	559.2625
$C_{17} H_{37} N_7 O_{14}$	563.2392
$C_{21} H_{45} N_{11} O_7$	563.3516
$C_{26} H_{55} N_{11} O_3$	569.4496
$C_{36} H_{73} O_4$	569.5513
$C_{33} H_{68} N O_6$	574.5065
$C_{32} H_{62} N_8 O$	574.5068
$C_{35} H_{73} N_3 O_3$	583.5650
$C_{35} H_{75} N_4 O S$	599.5656
$C_{18} H_{39} N_8 O_{15}$	607.2532
$C_{18} H_{39} N_8 O_{15}$	607.2532
$C_{23} H_{49} N_{11} O_8$	607.3776
$C_{29} H_{65} N_4 O_9$	613.4761
$C_{38} H_{79} N O_2 S$	613.5826
$C_{19} H_{41} N_9 O_{16}$	651.2666
$C_{30} H_{63} N_{11} O_5$	657.5014
$C_{37} H_{79} N_5 O_4$	657.6136
$C_{39} H_{85} N_6 O_2 S$	701.6413
$C_{28} H_{38} N_2 O_{19}$	706.209
$C_{22} H_{38} N_6 O_{20}$	706.2128
$C_{25} H_{50} N_6 O_{17}$	706.3273
$C_{35} H_{77} N_4 O_{12}$	745.5547
$C_{12} H_{18} N_3$	364.0992
*	495.5051
$C_{13} H_{12} N_6$	380.0719
*	398.3831
$C_{29} H_{52} N_3$	442.4143
*	467.4712
$C_{25} H_{41} N_3$	479.3007
*	495.5054
$C_{30} H_{57} N_4$	521.4437
*	530.4747
$C_{17} H_{37} N_7$	563.2392
*	618.5341
*	627.5951
*	643.5914
$C_{26} H_{59} N_4$	651.4025
*	2235.7092
*	2236.2046
*	3980.1887
*	3980.7107

*compounds for which the Software Mass Profile Professional. version 13.1.1 (Agilent Technologies) did not generate the molecular formula.
